# Aberrant plasma levels of circulating *miR-16, miR-107, miR-130a* and *miR-146a* are associated with lymph node metastasis and receptor status of breast cancer patients

**DOI:** 10.18632/oncotarget.3874

**Published:** 2015-04-19

**Authors:** Isabel Stückrath, Brigitte Rack, Wolfgang Janni, Bernadette Jäger, Klaus Pantel, Heidi Schwarzenbach

**Affiliations:** ^1^ Department of Tumor Biology, University Medical Center Hamburg-Eppendorf, Germany; ^2^ First Department of Obstetrics and Gynecology, Ludwig Maximilians University of Munich, Germany; ^3^ Department of Gynecology and Obstetrics, University Hospital Ulm, Germany

**Keywords:** cell-free miRs, chemotherapy, invasion, proliferation, tumor progression

## Abstract

Within the multicenter SUCCESS trial, we investigated the association of plasma microRNAs with different subtypes of invasive breast cancer.

Six miRs (*miR-16, miR-27a, miR-107, miR-130a, miR-132 and miR-146a)* were selected from microarray profiling and further validated in plasma of 111 breast cancer patients before and after chemotherapy and 46 healthy women by quantitative real-time PCR.

Plasma levels of *miR-16* (*p* = 0.0001), *miR-27a* (*p* = 0.039) and *miR-132* (*p* = 0.020) were higher in breast cancer patients before chemotherapy than healthy women. With the exception of miR-16, the increased levels of *miR-27a* (*p* = 0.035) and *miR-132* (*p* = 0.025) decreased after chemotherapy to those observed in healthy women. Levels of *miR-16* (*p* = 0.019), *miR-107* (*p* = 0.036), *miR-130a* (*p* = 0.027) and *miR-146a* (*p* = 0.047) were different between lymph node -positive and -negative patients, while the levels of *miR-130a* (*p* = 0.001) and *miR-146a* (*p* = 0.025) also differed between HER2-positive and -negative status. Estrogen-receptor negative tumors displayed higher concentrations of circulating *miR-107* than their counterparts (*p* = 0.035). However, overexpression of *miR-107* in MCF-7 cells did not downregulate estrogen receptor protein. Altered expression levels of *miR-107* influenced the migration and invasion behavior of MCF-7 and MDA-MB-231 cells.

Our data indicate differential concentrations of plasma *miR-16*, *miR-107*, *miR-130a* and *miR-146a* in different breast cancer subtypes, suggesting a potential role of these miRs in breast cancer biology and tumor progression.

## INTRODUCTION

In breast cancer the hormone receptor status is one of the most important predictive factor [1]. Since patients with positive hormone receptor status are eligible for endocrine adjuvant and palliative therapy, assessment of estrogen (ER) and progesterone (PR) receptor is routinely carried out. ER-positive tumors are classified as (highly hormone responsive) luminal A or (high proliferative and less hormone responsive) luminal B tumors. Targeted therapies that inhibit ER or ER-activated pathways include the selective ER modulator tamoxifen and the aromatase inhibitor arimidex. Anti-HER2 (human epidermal growth factor receptor 2) therapies include the administration of trastuzumab and lapatinib. Unfortunately, in spite of receptor positivity, many tumors can develop resistance to therapy, and the expression pattern of ER, PR and HER2 may change over time as tumors progress and metastasize [2].

MicroRNAs (miRs) are evolutionary conserved, small non-coding RNA molecules consisting of approximately 22 nucleotides. MiRs inhibit the gene expression post-transcriptionally by binding specifically to the 3′untranslated-region (UTR) of their target mRNA. Gene silencing can occur through the translational inhibition or cleavage of their target mRNAs depending on the complementary sequence between binding sites of the specific mRNA and miR [3]. Computational analyses indicate that one miR has binding affinity to hundreds of different mRNAs and hence, miRs are involved in the regulation of various cellular processes, such as development, differentiation and proliferation [4].

As miR loci frequently map to fragile chromosomal regions harboring DNA amplifications, deletions or translocations, their expression is often deregulated during tumorigenesis, contributing to tumor progression [5-8]. In this context, they may act as so-called oncomiRs by targeting tumor suppressor genes or as tumor suppressor miRs by inhibiting oncogenes [9]. MiRs are released into the blood circulation by apoptotic and necrotic cells or active secretion [10], and exist either cell-freely, associated with Argonaut proteins, or in exosomes in the blood circulation [11]. To date, numerous miRs have been identified, those transcript levels were dysregulated in the blood of breast cancer patients [12, 13].

In the present study, we focused our analyses on circulating *miR-27a*, *miR-107*, *miR-130a*, *miR-132*, *miR-146a* and *miR-16*, those dysregulated transcript levels were detected in patients with invasive breast cancer by microarray profiling. *MiR-27a*, that promotes angiogenesis by mediating endothelial differentiation of breast cancer stem like cells [14], has been associated with poor overall survival of breast cancer patients and suggested to be a valuable marker for tumor progression [15]. *MiR-107* targets numerous transcripts including estrogen-responsive gene clusters, and its intracellular levels are affected by estrogen [16]. *MiR-130a* contributes to the growth inhibition induced by retinoic acid that is used as a chemopreventive agent for breast cancer [17]. Overexpression of *miR-132* has been reported to inhibit proliferation of breast cancer cells and their colony formation [18]. Transduction of *miR-146a* may down-regulate expression of epidermal growth factor receptor (EGFR), and inhibit invasion and migration of breast cancer cells [19]. To date, *miR-16* has been often used as reference miR to normalize miR data, because this miR showed the smallest variation in the populations, but *miR-16* has also been elevated in osteoclast differentiation associated with bone metastasis burden [10, 20].

The aim of this study was to investigate whether the dysregulated levels of circulating *miR-27a*, *miR-107*, *miR-130a*, *miR-132*, *miR-146a* and *miR-16* are associated with the receptor status of breast cancer. Screening for plasma miRs could provide valuable non-invasive information on the luminal subtype of breast cancer. The effect of adjuvant chemotherapy on the miR levels was also investigated.

## RESULTS

### Patients

**Figure 1:**
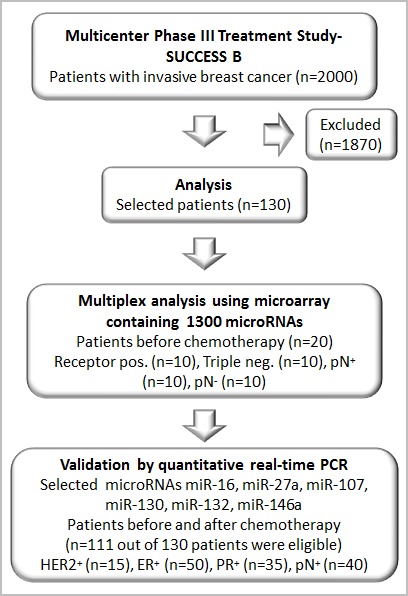
Consort diagram showing the number of patients analyzed from the multicenter study (SUCCESS B)

For this retrospective study, we chose 130 patients with invasive breast cancer from the SUCCESS study based on their receptor status, to assemble a collective that showed a well-balanced distribution of established risk factors including hormone receptor status, lymph node status, tumor stages, and grading. Of this cohort, 42% of the patients were triple-negative and/or had lymph node metastases (Figure 1). This highly selected cohort does not represent the population of the SUCCESS study (www.success-studie.de). Blood plasma samples were taken from these patients about 1 or 2 months after surgery before initiation of chemotherapy and additionally, collected after chemotherapy. For our miR analyses, 111 out of 130 patients were eligible. All patients analyzed had histologic proven epithelial cancer. Table 1 summarizes the clinical and histopathologic parameters of the breast cancer patient cohort.

**Table 1:** Patient characteristics and histopathological parameters at the time after surgery

Parameters	Patients (%)
**Breast cancer patients**	
**Total**	**111 (100)**
**Age**	**56 years (range 33-76 years)**
**Tumor stage**	
pT1	59 (53)
pT2-4	52 (47)
**Lymph node metastasis**	
N0	71 (64)
N1-3	40 (36)
**Grading**	
II	40 (36)
III	71 (64)
**Estrogen receptor status**	
positive	50 (45)
negative	61 (55)
**Progesterone receptor status**	
positive	35 (32)
negative	76 (68)
**HER2 status**	
positive	15 (14)
negative	96 (86)
**Triple negative**	
Triple neg.	47 (42)
other receptor status	64 (58)
**Healthy women**	
**Total**	**46 (100)**
**Age**	**63 years (range 50-85 years)**

### MiR profiling using a blood-based microarray

**Figure 2:**
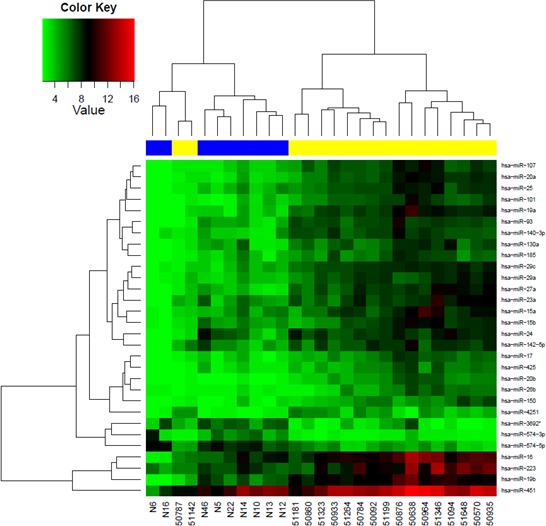
MiR profiling using a blood-based microarray Hierarchical cluster heat map of miR microarray was performed using microfluid biochips containing 1300 different miRs and postoperative plasma of 20 breast cancer patients before chemotherapy and 10 healthy women. The colored representation of samples and probes is ordered by their similarity with a dendogram on top (clustering of samples) and on the right side (clustering of probes).

For blood-based miR profiling, microfluid biochips containing 1300 different miRs were used to quantify the expression of miRs in the plasma samples of 20 breast cancer patients and 10 healthy women. To detect differentially regulated miRs, the quotation of median, paired Student's *t*-test and limma test were assessed. Dysregulated miRs were detected by the highest absolute value of logarithmized bold changes in comparison of breast cancer patients with healthy women. The estimated raw *p*-values were adjusted for multiple testing, to control the false discovery rate. Table 2 shows 30 most differentially expressed miRs with the adjusted *p*-values as determined by *t*-test and limma test. In addition, the normalized median values of healthy women and breast cancer patients are listed, and the comparison of these values in both cohorts indicates the down- or upregulated miRs. A similarity matrix was generated containing all pairwise similarities of the plasma samples of breast cancer patients and healthy women. To detect potential clusters in rows (transcripts) and columns (samples) of the normalized expression matrix, hierarchical clustering was carried out (Figure 2). Based on the highest overall variability derived from these array data (Table 2), we selected *miR-16, miR-27a, miR-107* and *miR-130a* for further validation studies. In addition, we also quantified *miR-132* and *miR-146a*, because of their particular role in breast cancer [18, 21]. *MiR-1207* was chosen as reference miR, because it displayed the smallest data variations in the miR profiling.

**Table 2:** Differentially regulated microRNAs in healthy individuals versus breast cancer patients

microRNA	normalized median values		t-test adjusted p-value	limma test adjusted p-value
	healthy	BCa patients		
hsa-miR-19a	8.4	197.0	0.0170	0.0001
**hsa-miR-27a**	**8.6**	**168.7**	**0.0026**	**0.0001**
hsa-miR-20a	9.3	149.7	0.0022	0.0001
hsa-miR-101	6.2	93.1	0.0151	0.0001
hsa-miR-29c	14.2	198.7	0.0003	0.0001
hsa-miR-574-5p	309.2	25.0	0.0064	0.0001
hsa-miR-3692*	92.9	7.9	0.0121	0.0001
**hsa-miR-107**	**10.2**	**111.6**	**0.0061**	**0.0001**
hsa-miR-140-3p	11.5	120.9	0.0026	0.0001
**hsa-miR-130a**	**8.6**	**90.1**	**0.0079**	**0.0017**
hsa-miR-29a	12.4	124.3	0.0014	0.0001
hsa-miR-15a	18.2	177.0	0.0400	0.0035
hsa-miR-26b	5.7	42.5	0.0081	0.0021
hsa-miR-17	6.9	51.1	0.0066	0.0004
hsa-miR-142-5p	20.7	149.4	0.0136	0.0004
hsa-miR-25	18.0	127.8	0.0026	0.0006
hsa-miR-223	109.5	773.5	0.0478	0.0114
hsa-miR-24	29.9	201.6	0.0432	0.0118
**hsa-miR-16**	**154.9**	**1038.9**	**0.0470**	**0.0030**
hsa-miR-20b	5.3	34.5	0.0050	0.0001
hsa-miR-23a	26.3	169.2	0.0191	0.0010
hsa-miR-93	21.8	140.3	0.0136	0.0003
hsa-miR-19b	94.7	606.2	0.0129	0.0021
hsa-miR-451	1636.5	10409.9	0.0232	0.0251
hsa-miR-574-3p	37.8	6.3	0.1391	0.0001
hsa-miR-150	5.4	32.0	0.0149	0.0006
hsa-miR-15b	25.2	144.4	0.0149	0.0138
hsa-miR-185	12.2	67.7	0.0066	0.0013
hsa-miR-425	6.9	37.7	0.0088	0.0014
hsa-miR-4251	5.6	29.6	0.0006	0.0001

### Profiling of cell-free *miR-16, miR-27a, miR-107, miR-130a, miR-132* and *miR-146a* in the postoperative plasma of breast cancer patients before and after chemotherapy

Before we quantified the relative transcript levels of our panel of selected miRs in the plasma of 111 patients with invasive breast cancer before and after chemotherapy and 46 age-matched healthy women by quantitative real-time PCR, we measured the total small RNA in the plasma of both cohorts. The plasma RNA levels were higher in patients before chemotherapy than in healthy women (*p* = 0.0001). The significant increased plasma RNA concentrations detected before chemotherapy significantly decreased after chemotherapy (*p* = 0.001), but were still higher than the plasma levels in healthy women (*p* = 0.021, Supplementary Figure S1).

**Figure 3:**
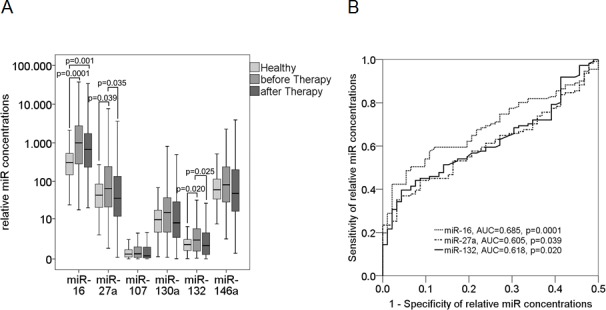
Plasma levels of circulating miR-16, miR-27a, miR-107, miR-130a, miR-132 and miR-146a in breast cancer patients before and after chemotherapy The box plot compares the miR concentrations in plasma of healthy women (*n* = 46) with breast cancer patients before (*n* = 111) and after chemotherapy (*n* = 111). The relative expression levels were determined by the low cycle threshold (Ct) values. As determined by Mann-Whitney-U and Wilcoxon test, the significant *p*-values of statistical evaluations are indicated **A.**. ROC analyses show the profiles of sensitivity and specificity of *miR-16, miR-27a* and *miR-132* to differentiate healthy individuals from breast cancer patients before chemotherapy **B.**.

Then, we quantified cell-free *miR-16, miR-27a, miR-107, miR-130a, miR-132* and *miR-146a* by Taqman PCR. As shown in the box plot of Figure 3A, the postoperative plasma levels of *miR-16* (*p* = 0.0001, Mann-Whitney-U test), *miR-27a* (*p* = 0.039) and *miR-132* (*p* = 0.020) were significantly higher in breast cancer patients before chemotherapy than in healthy women. The AUC values of *miR-16, miR-27a* and *miR-132* were 0.685 (*p* = 0.0001), 0.605 (*p* = 0.039) and 0.618 (*p* = 0.020), respectively, showing the significant difference of the miR levels between patients before chemotherapy and healthy women (Figure 3B). In contrast, the concentrations of *miR-107, miR-130a* and *miR-146a* were similar between the two cohorts. After chemotherapy, the elevated levels of *miR-27a* (*p* = 0.035, Wilcoxon test) and miR-132 (*p* = 0.025) decreased to similar levels as observed in healthy women, whereas the levels of *miR-16* remained significantly increased (Figure 3A).

No association of the different treatment arms with the miR levels after chemotherapy was observed. Unfortunately, in the multicenter SUCCESS trial data on endocrine therapy of only 43 patients were available. Of these patients, 17 and 26 patients got tamoxifen and arimidex, respectively. We did not carry out a statistical evaluation, because the patient cohort was too small.

### Association of the plasma levels of *miR-16, miR-107, miR-130a* and *miR-146a* with lymph node and receptor status

We compared the relative concentrations of circulating *miR-16, miR-27a, miR-107, miR-130a, miR-132* and *miR-146a* in the postoperative plasma of 111 breast cancer patients before chemotherapy with the clinical and histopathological risk factors of these patients. To determine the diagnostic value of these miRs, the Mann-Whitney-U test was applied. The Supplementary Table S1 summarizes mean and median values, 95% confidence intervals (CI) and p values of the miR variables in the different patient subgroups.

**Figure 4:**
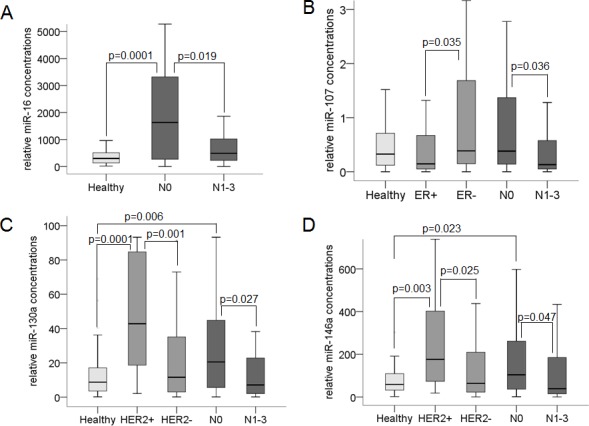
Association of cell-free miR-16, miR-107, miR-130a and miR-146a with lymph node and receptor status The box plots compare the plasma concentrations of *miR-16* in lymph node-negative (*n* = 71) with those in lymph node-positive patients (*n* = 40) **A.**, of *miR-107* in estrogen-positive (*n* = 50) with those in estrogen-negative patients (*n* = 61) and in lymph node-negative (*n* = 71) with those in lymph node-positive patients (*n* = 40) **B.**, and of *miR-130a* **C.** and *miR-146a* **D.** in HER2-positive (*n* = 15) with those in HER2-negative patients (*n* = 96) and in lymph node-negative (*n* = 71) with those in lymph node-positive patients (*n* = 40). As determined by the Mann-Whitney-U test, the significant *p*-values of statistical evaluations are indicated.

Albeit the significance was only marginal, the plasma levels of *miR-16* (Figure 4A, *p* = 0.019), *miR-107* (Figure 4B, *p* = 0.036), *miR-130a* (Figure 4C, *p* = 0.027), and *miR-146a* (Figure 4D, *p* = 0.047) could differ between lymph-node negative and positive patients. The elevated plasma levels of *miR-16* (Figure 4A, B *p* = 0.0001), *miR-130a* (Figure 4C, *p* = 0.006) and *miR-146a* (Figure 4D, *p* = 0.023) detected in lymph-node negative patients decreased in lymph-node positive patients to similar levels as observed in healthy women, suggesting that these miRs play rather a role in early breast cancer.

In addition, the plasma levels of *miR-107* could distinguish between ER-negative and -positive status and were significantly elevated in ER-negative patients (Figure 4B, *p* = 0.035). The plasma concentrations of *miR-130a* (Figure 4C, *p* = 0.001) and *miR-146a* (Figure 4D, *p* = 0.025) could differ between HER2-negative and -positive tumors, and were significantly elevated in HER2-positive patients.

### Expression levels of *miR-16, miR-27a, miR-107, miR-130a, miR-132* and *miR-146a* in MCF-7 and MDA-MB-231 cells

**Figure 5:**
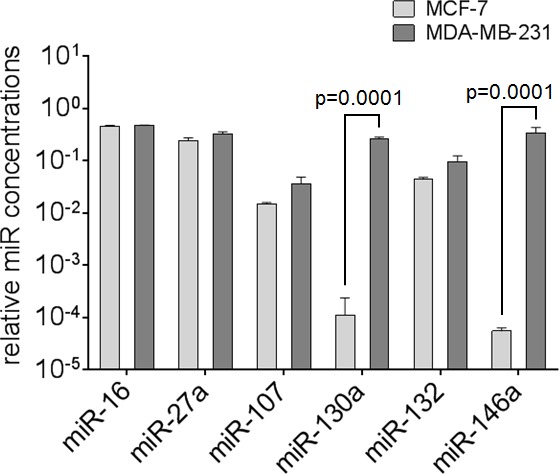
Expression levels of circulating miR-16, miR-27a, miR-107, miR-130a, miR-132 and miR-146a in breast cancer cell lines The bar chart compares the miR concentrations in non-invasive MCF-7 cells with those in invasive MDA-MB-231. The relative expression levels were determined by the low cycle threshold (Ct) values. The significant *p*-values as determined by the one-way ANOVA test and standard deviations from triplicate experiments are indicated.

The relative expression levels of our miR panel were determined in non-invasive, ER-positive breast adenocarcinoma MCF-7 cells and invasive, ER-negative MDA-MB-231 cells [22] by quantitative real-time PCR (Figure 5). These breast adenocarcinoma cell lines showed a heterogeneous miR pattern. The transcript levels of *miR-16, miR-27a, miR-107* and *miR-132* were similar in both cell lines, while *miR-130a* (*p* = 0.0001) and *miR-146a* (*p* = 0.0001) were significantly higher expressed in MDA-MB-231 cells than MCF-7 cells.

### *MiR-107* does not affect the protein expression of ER

Since our findings suggest that *miR-107* is associated with ER-negative breast cancer, we examined whether ER is a target of *miR-107*. Therefore, we screened the miR databases DIANA-microT-CDS [23], microRNA.org [24] and TargetScanHuman [25], and detected two potential binding sites of *miR-107* in the 3′UTR of ER (Supplementary Figure S2A). To examine whether expression of ER is regulated by *miR-107*, we performed transfections of non-invasive, ER-positive MCF-7 cells. We transiently transfected MCF-7 cells with mimics and inhibitors of *miR-107* and with an expression plasmid encoding for *miR-107*. The mimics are double-stranded RNA molecules which mimic the endogenous, mature *miR-107*, whereas the inhibitors are single-stranded, modified RNA molecules which after transfection, specifically binds to mimics and endogenous *miR-107*, and inhibits its function. The transfection with scramble miR served as a negative control. We confirmed the overexpression of *miR-107* in the transfected cells by real-time PCR (data not shown). The data of the quantitative real-time PCR using gene-specific primers and Western blots using protein-specific antibodies specific for ER showed no effect of the mimics and inhibitors on the ER RNA and protein levels, respectively (Figure S2B and S2C). These findings suggest that *miR-107* is not involved in the repression of ER in MCF-7 cells.

### *MiR-130a* affects cell proliferation in MCF-7 cells

The effects of *miR-16, miR-107* and *miR-130a* were also investigated on apoptosis and proliferation of MCF-7 and MDA-MB-231 cells. We transiently transfected these cells with mimics and inhibitors of *miR-16, miR-107* and *miR-130a*. To induce apoptosis, the transfected cells were treated with topoisomerase I inhibitor camptothecin. FACS analyses did not show any effect on camptothecin mediated apoptosis by overexpression or inhibition of these miRs in the cell lines (Supplementary Figure S3).

**Figure 6:**
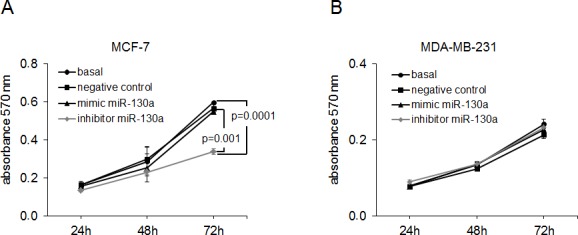
MiR-130a affects proliferation of MCF-7 cells MCF-7 **A.** and MDA-MB-231 **B.** cells were transfected with mimic or inhibitor of *miR-130a* and treated with MTT. Transfection with scramble miR served as a negative control. Cell proliferation after overexpression and inhibition of *miR-130a* for 24 h, 48 h and 72 h. Inhibition of endogenous *miR-130a* reduced cell proliferation in MCF-7 cells as compared with basal MCF-7 cells and the negative control. The significant *p*-value as determined by the one-way ANOVA test and standard deviations from triplicate experiments are indicated.

Additionally, the effects of *miR-16, miR-107* and *miR-130a* on cell proliferation were evaluated by a MTT assay. However, we only observed an effect on cell proliferation by *miR-130a* in MCF-7 cells, but not by *miR-16* and *miR-107* and in MDA-MB-231 cells. As shown in Figure 6, transfection of *miR-130a* inhibitor reduced the cell proliferation in non-invasive MCF-7 cells compared with analogous basal cells (*p* = 0.0001), whereas surprisingly, administration of *miR-130a* mimic had no effect on cell proliferation in this cell line. This could be explained that the endogenous expression level of *miR-130a* is too high in MCF-7 cells (albeit much lower than in MDA-MB-231), so that exogenously introduced *miR-130a* is not effective. Even using different concentrations of mimics and inhibitors for transfection of MCF-7 and MDA-MB-231 cells similar results were observed (Supplementary Figure S4).

### *MiR-107* affects migration and invasion of MCF-7 and MDA-MB-231 cells

**Figure 7:**
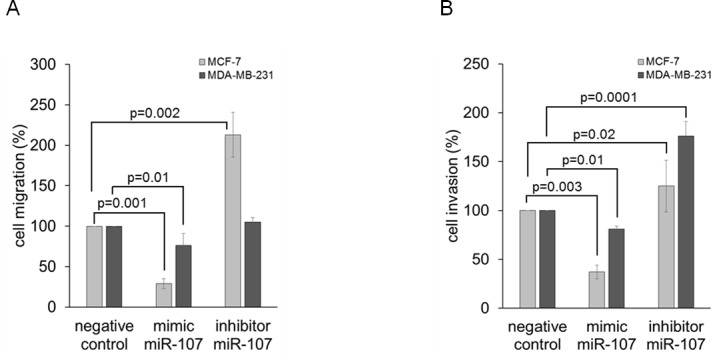
MiR-107 affects migration and invasion of MCF-7 and MDA-MB-231 cells Cell migration **A.** and invasion **B.** of MCF-7 and MDA-MB-231 cells were analyzed by transwell assays. Cells were transfected with mimic or inhibitor of *miR-107* prior to serum starvation for 24 h. Cells were added to the upper transwell chamber that was uncoated for the migration assay and BME-coated for the invasion assay, and allowed to migrate or invade for 24 h. Migrated or invaded cells were stained with Calcein-AM, and fluorescence (excitation: 485 nm, emission: 535 nm) was measured. Overexpression of *miR-107* decreased migration and invasion in MCF-7 and MDA-MB-231 cells as compared with the negative control (transfection with scramble miR). Inhibition of endogenous *miR-107* increased invasion in both cell lines, and migration in MCF-7 cells but not in MDA-MB-231 cells. The significant *p*-values as determined by the one-way ANOVA test and standard deviations from triplicate experiments are indicated.

Our current findings show that low levels of *miR-107* correlated with a positive ER (*p* = 0.035) and lymph node status (*p* = 0.036). To examine whether *miR-107* has an inhibitory effect on cell migration and invasion, respective assays were carried out using non-invasive MCF-7 and invasive MDA-MB-231 cells. As shown in Figure 7A, the overexpression of *miR-107* significantly reduced the ability of MCF-7 (*p* = 0.001) and MDA-MB-231 (*p* = 0.01) cells to migrate compared with the negative control. The inhibition of *miR-107* could increase migration of MCF-7 (p = 0.002), but no significant impact of MDA-MB-231 cells could be observed. Administration of *miR-107* mimic also reduced the invasiveness of MCF-7 (*p* = 0.003) and MDA-MB-231 (*p* = 0.01) cells (Figure 7B). Transfection of *miR-107* inhibitor stimulated the invasion of MCF-7 (*p* = 0.02) and MDA-MB-231 (*p* = 0.0001) cells through matrigel-coated transwell membranes. These findings show that alterations of the *miR-107* level could change the migration and invasion behavior of cells. Therefore, *miR-107* could play an important role in tumor progression.

## DISCUSSION

Based on our microarray data and their particular characteristics, we quantified six miRs (*miR-16, miR-27a, miR-107, miR-130a, miR-132* and *miR-146a*) in postoperative plasma of breast cancer patients before and after chemotherapy and healthy women. As far as we know, only *miR-16* and *miR-146a* of our panel of selected miRs have been quantified in the plasma or serum of breast cancer patients by other studies. The quantification of circulating *miR-16* was often used as a normalization control [10]. The levels of *miR-146a* were measured in the plasma of Indian breast cancer patients and were significantly higher than in healthy women, indicating the diagnostic potential of this miR in breast cancer [26].

In our study, the plasma levels of *miR-27a* and *miR-132* were increased in breast cancer patients before chemotherapy and decreased to normal (wild type) levels after chemotherapy, whereas the increased levels of *miR-16* were not affected by chemotherapy. No correlation of the plasma levels of these miRs with different patient treatment arms could be observed. The levels of *miR-16, miR-107, miR-130a* and *miR-146a* were significantly higher in lymph-node negative patients than in their counterparts. Moreover, the concentrations of *miR-107* were significantly increased in ER-negative patients compared with ER-positive patients, suggesting a suppressive effect on ER expression *in vivo*. However, our experimental studies on MCF-7 cancer cells showed that *miR-107* could not downregulate ER protein expression *in vitro*. Finally, the plasma levels of *miR-130a* and *miR-146a* correlated with the HER2 status and were significantly increased in HER2-positive patients. Inhibition of *miR-130a* reduced the cell proliferation in non-invasive MCF-7 cells. Overexpression of *miR-107* decreased migration and invasion of MCF-7 and MDA-MB-231 cells, while inhibition of *miR-107* stimulated invasion of MCF-7 and MDA-MB-231 cells and migration of MCF-7 cells.

Discrepant data on *miR-16* exist in numerous publications that describe this miR as oncogene, tumor suppressor gene or reference miR [27-29]. *MiR-16* is most frequently used as endogenous control for data normalization, because this miR is highly expressed in plasma or serum, and has been described as being relatively invariant across diverse blood samples [30]. On the other hand, *miR-16* expression has also been reported to be a potential therapeutic target and clinical biomarker of bone metastasis, because it is elevated in osteoclast differentiation and bone metastasis [20]. In our study, the levels of *miR-16* were significantly increased in lymph node-negative patients and decreased to normal levels in patients with lymph node metastases. These findings point to a wavelike transcription of *miR-16* during tumor development and progression, but also to a varying expression pattern of *miR-16* in different tumors. However, to interpret more exactly the different transcript levels of *miR-16*, large patient populations should be investigated and most importantly, a standardized reference for normalization of the data should be established. To better comprehend the different features of *miR-16*, we looked for their mRNA targets, and found that *miR-16* controls the intrinsic apoptosis pathway in breast cancer cell lines. Overexpression of *miR-16* downregulated expression of the apoptosis inhibitor Bcl-2 at the protein level in MCF-7 cells, whereas suppression of *miR-16* increased expression of Bcl-2 in MDA-MB-231 cells [31]. However, in our present study we could not observe such an apoptotic effect in MCF-7 and MDA-MB-231 cells. It is difficult to explain these discrepant data that could be rather caused by different experimental procedures. Moreover, we detected that in contrast to the other miRs of our panel, chemotherapy did not affect the increased levels of *miR-16* in postoperative plasma of the breast cancer patients. This could be explained by inflammatory processes which release increasingly *miR-16* into the blood circulation.

In our study, the plasma levels of circulating *miR-107* were significantly higher in lymph node-negative and ER-negative tumors than in their respective positive counterparts. Though, the association of increased levels of *miR-107* with ER-negative tumor is not caused by a downregulation of ER by *miR-107*, as shown by our *in vitro* data on MCF-7 cells. Nevertheless, the detection of decreased amounts of *miR-107* in our subpopulation of ER-positive patients is supported by a previous study. This study reported an inhibitory effect of estrogen on the intracellular levels of *miR-107* and its down-regulation in estrogen treated MCF-7 cells [16]. These and our present results point to that estrogen may negatively regulate the gene expression of *miR-107*. Moreover, we found that inhibition of *miR-107* may stimulate migration of MCF-7 cells and invasion of MCF-7 and MDA-MB-231 cells. Overexpression of *miR-107* inhibits the migratory and invasive ability of both cell lines. Our findings are supported by investigations in other tumor types such as glioma or cervical cancer. Enhanced *miR-107* expression significantly inhibited invasion of glioma stem cells and reduced matrix metalloproteinase-12 expression [32]. It was shown for cervical cancer cells that *miR-107* directly targeted MCL1 and activated ATR/Chk1 pathway to inhibit proliferation, migration and invasiveness of cervical cancer cells. Overexpression of *miR-107* resulted in a significant reduction of migratory and invasive potential of the cells. Inhibition of *miR-107* led to significant increase in invasive potential of HeLa cells [33]. As far as we know, only one study has investigated the role of *miR-107* in breast cancer cells. It was reported that *miR-107* negatively regulated the expression of CDK8, that is involved in the regulation of cell cycle and cell growth, and inhibited the proliferation and migration of MDA-MB-231 cell line [34]. Our results additionally demonstrate the potential role of *miR-107* in migration and invasion of MCF-7 cells by overexpression and inhibition of this miR. These data may explain the high *miR-107* transcript levels detected in patient with negative lymph node stage.

Moreover, we detected that increased plasma levels of *miR-130a* and *miR-146a* correlated with lymph node-negative and HER2-positive status of the patients. In contrast to our findings showing that *miR-130a* affects cell proliferation, but has no apoptotic effect, *miR-130a* was described as an inhibitor of apoptosis. Its expression may be directly regulated by the oncogene product c-Myc. Based on the frequent amplification of c-Myc in human cancers, high levels of c-Myc protein may provoke high expression of *miR-130a*, which may prevent apoptosis of cancer cells [17]. *MiR-146a* binds to the 3′UTR of BRCA1 and down-regulates its expression. This down-regulation was accompanied by an increased proliferation and a reduced homologous recombination rate, two processes controlled by BRCA1 [21]. Transfection of *miR-146a* into MDA-MB-231 inhibited invasion and migration *in vitro* and breast cancer metastasis [19]. Further work is needed to better understand the complex role of *miR-130a* and *miR-146a*.

In our panel of miRs, chemotherapy had an effect on the plasma levels of *miR-27a* and *miR-132*. The elevated amounts before chemotherapy decreased to normal levels after chemotherapy. As far as we know, there are no analyses on cell-free *miR-27a* and *miR-132* in the blood circulation of breast cancer patients. Functional analyses showed that *miR-27a* promotes angiogenesis by mediating endothelial differentiation [14], and its expression in primary tumor tissues was associated with poor overall survival of breast cancer patients [15]. Conversely, the expression of *miR-132* was found to be significantly deregulated in ductal carcinoma in situ (DCIS) [18].

In conclusion, clinically relevant, quantitative changes in the transcript levels of miRs can be detected in the blood circulation of cancer patients. Their screening in plasma of patients with invasive breast cancer may provide information on the aberrant signaling pathway that could be blocked by the chosen targeted therapy. The clinical use of these “liquid biopsies” could, therefore, contribute to the understanding of the molecular mechanisms underlying breast cancer development and progression. Our findings demonstrate the modulations of circulating *miR-16, miR-27a, miR-107, miR-130a, miR-132* and *miR-146a* in the plasma of patients with invasive breast cancer and their association with specific breast cancer subtypes and biological behavior. Prospective studies on larger cohorts of patients are required to substantiate their diagnostic role.

## MATERIALS AND METHODS

### Study populations

Within a multicenter study (SUCCESS), which includes 251 German centers, postoperative blood plasma samples were collected from patients with invasive breast cancer. Plasma samples of 111 eligible patients participating at this study were analyzed before (July 2008 to May 2011) and after chemotherapy (November 2008 to September 2011, Figure 1). In addition, plasma samples were collected from 46 age-matched healthy women with no history of cancer and in good health based on self-report. All patients and healthy controls gave their informed consent. The study was approved by the ethic committee of the Ludwig-Maximilians University Munich and conducted in accordance with the Declaration of Helsinki.

The tumor stage at primary diagnosis of the patients was classified according to the revised American Joint Committee on Cancer tumor-node-metastasis classification. Histopathologic grading of the primary tumors was conducted according to the Bloom-Richardson system. For the diagnosis of lymph node metastasis, single embedded lymph nodes were screened up to 3 levels. External beam radiation therapy was administered in all patients treated with breast conservation. Chest wall irradiation following mastectomy was conducted in patients with more than 3 involved lymph nodes or T3 and T4 stage tumors. The adjuvant therapy included 3 cycles of 5-Fluorouracil 500 mg/m² i.v. body surface area and Epirubicin 100 mg/m² i.v. and Cyclophosphamide 500 mg/m² i.v., (FEC100), each administered on day 1, repeated on day 22, subsequently followed by 3 cycles of Docetaxel 75 mg/m² body surface area i.v., and Gemcitabine 1000 mg/m² i.v. (30 min infusion), administered on day 1, followed by Gemcitabine 1000 mg/m² i.v. (30 min infusion) on day 8, repeated on day 22. After chemotherapy, women with hormone receptor- positive disease received endocrine treatment (tamoxifen and arimidex). As determined by immunohistochemistry (IHC), a cut-off level of more than 10% was used for positive hormone receptor status. For the expression of HER2, the DAKO score from 0 to 3b was determined by IHC (+++) and FISH (+) analyses, according to the recommendations of American Society of Clinical Oncology. Women with HER2-positive disease received a therapy with trastuzumab.

### MicroRNA profiling

MicroRNA profiling was performed with plasma samples derived from 20 breast cancer patients and 10 healthy women. Small RNA was isolated from 600 μL plasma using NucleoSpin miRNA Plasma Kit (Macherey-Nagel, Düren, Germany) and according to the manufacturer's protocol. Blood-based microarray profiling was performed with SurePrintG3 Human miRNA (8x60K) microarrays (Agilent Technologies; Febit Biomed GmbH, Heidelberg, Germany) containing 1300 human and viral miRs derived from Sanger miRBase release 16.0 (April 2011). Samples were labeled with pCp-Cy3 using T4 ligase and hybridized on 8x60K format Agilent human miRNA array. Data were acquired using Agilent Feature Extraction software version 10.7.3.1.

### Extraction of small RNA and conversion into cDNA

Small RNA was extracted from 300 μL plasma using the NucleoSpin miRNA Plasma Kit (Macherey-Nagel) and according to the manufacturer's instructions. For extraction efficiency, 20 fmol of synthetic non-human *cel-miR-39* was added as an exogenous spike in control. The RNA was quantified on a NanoDrop ND-1000 Spectrophotometer (Thermo Scientific, Wilmington, Delaware, USA) and stored at −80°C until they were reverse transcribed into cDNA.

Reverse transcription was performed by the TaqMan MicroRNA Reverse Transcription Kit (Life Technologies, New York, USA). The 10 μL-reverse transcription reaction contained 0.1 μL 100 mM dNTPs, 0.66 μL MultiScribe Reverse Transcriptase (50 U/μL), 1 μL 10× Reverse Transcription Buffer, 0.13 μL RNase Inhibitor (20 U/μL), 2 μL 5x TaqMan RT Primer, nuclease-free water and 2 μL RNA. The reaction was carried out at 16°C for 30 min., 42°C for 30 min. and 85°C for 5 min. on a MJ Research PTC-200 Peltier Thermal Cycler (Global Medical Instrumentation, Ramsey, Minnesota, USA). The cDNA samples were stored at −20°C until further use.

### Quantitative real-time PCR of *miR-16, miR-27a, miR-107, miR-130a, miR-132* and *miR-146a*

For quantitative real-time PCR, the miR-specific TaqMan MicroRNA Assays (Life Technologies) for *miR-1207* (reference miR), *miR-16, miR-27a, miR-107, miR-130a, miR-132* and *miR-146a* were used. In a 10 μL-reaction, 1 μL cDNA were mixed with 5 μL TaqMan Gene Expression Master Mix and 0.5 μL 20x miR-specific TaqMan MicroRNA Assay on a twin-tec real-time PCR plate (Eppendorf, Hamburg, Germany). The quantitative real-time PCR reaction was performed at 95°C for 10 min. and for 40 cycles at 95°C for 15 s and 60°C for 60 s on a Mastercycler Realplex (Eppendorf).

As there is no consensus concerning the normalization of circulating miRs, we chose *miR-1207* as reference to normalize our miR data, because this miR showed the smallest coefficient variation of the populations as measured by the blood-based microarray (0.262). In the plasma samples of breast cancer patients, we calculated a mean value of 29.67 (SD = 1.75) before therapy and of 29.42 (SD = 1.79) after therapy. The mean value in healthy controls was 30.51 (SD = 1.46). These findings show the relative constant expression of *miR-1207* in the different cohorts. The obtained data of the miR expression levels were calculated and evaluated by the ΔCt method as follows: ΔCt = mean value Ct (reference *miR-1207*) - mean value Ct (miR of interest). The relative expression of the miR of interest corresponded to the 2^(ΔCt) value.

To avoid quantifying our miR panel in hemolytic plasma samples that may influence our results, we excluded such plasma samples that color changed in red or dark red. In addition, we also examined plasma samples for contamination by hemolysis using *miR-451* and *miR-23a*. As described by Blondel et al., if the ratio between these miRs is higher than 5, it is an indicator of possible erythrocyte miR contamination, and a ΔCt of 7–8 or more indicates a high risk of hemolysis affecting the data obtained [35]. Our data for our measurements of the ratio between *miR-451* and *miR-23a* showed a mean value of 3.19 with a standard deviation of 1.27, a median value of 3.73 and a range between 0.32 and 5.26, indicating that the plasma samples were not contaminated.

### Transient transfection of *miR-16, miR-107* and *miR-130a*

The breast adenocarcinoma cell lines MCF-7 and MDA-MB-231 (ATCC) were cultured in DMEM (Invitrogen, Karlsruhe, Germany) supplemented with 10% FCS (fetal calf serum; PAA Laboratories, Cölbe, Germany) and 2 mM L-glutamin (Life Technologies) under standard conditions (37°C, 10% CO_2_, humidified atmosphere). Using Multiplex Cell Authentication (Multiplexion, Heidelberg, Germany) they were authenticated within six months of use. 3*10^5^ of cells were seeded on 6-well plates (NUNC, Roskilde, Denmark) and transfected with 1 μg of an expression plasmid encoding for *miR-107* or the double-stranded miScript miRNA mimics *miR-16, miR-107* and *miR-130a* at final concentration of 10 nM (Qiagen, Hilden, Germany) or the analogous single-stranded miScript miRNA inhibitors at final concentration of 50 nM (Qiagen) with 2 μL X-tremeGENE HP DNA Transfection Reagent (Roche Diagnostics, Mannheim, Germany). As a negative control served the transfection with miScript scramble at final concentration of 50 nM (Qiagen). For titration experiments the cells were transfected with *miR-16*, *miR-107* and *miR-130a* mimics at final concentration of 5, 10, 20, or 30 nM and with inhibitors at final concentration of 25, 50, 100, or 150 nM.

The expression (pcDNA3.1) plasmid encoding for *miR-107* was constructed by annealing the DNA sequences of 5′-TGCAGAATTCCTCTCTGCTTTCAG CTTCTTTACAGTGTTGCCTTGTGGCATGGAGTTCA-3′ and 5′-AGTATCTCGAGTCTGTGCTTTGATAGCCC TGTACAATGCTGCTTGAACTCCATGCCACA-3′. The 5′ overhanging ends were filled in by the Klenow fragment DNA polymerase (Thermo Scientific). The double-stranded DNA was cut by the restriction enzymes EcoRI and XhoI (New England Biolabs, Frankfurt, Germany) and cloned into the multiple cloning site of the pcDNA 3.1 vector (Life Technologies). The cloned expression plasmid was verified by DNA sequencing.

### Quantitative real-time PCR and western blot of ER expression

After 48 hour incubation of untreated and transfected MCF-7 and MDA-MB-231 cells, total RNA and protein were extracted using peqGOLD TriFast (Peqlab, Erlangen, Germany) according to the manufacturer`s instructions.

To determine mRNA expression of ER, a total of 200 ng RNA from basal and transfected cells was reverse transcribed using the First strand cDNA synthesis kit (Thermo Scientific). The mRNA expression levels were subsequently quantified by real-time PCR using the Maxima SYBR Green/ROX qPCR Master Mix (Thermo Scientific) and the following primers for ER (forward: 5′-GCATTCTACAGGCCAAATTCA-3′ and reverse: 5′-TCCTTGGCAGATTCCATAGC-3′). GAPDH (forward: 5′-CCTGCACCACCAACTGCTTAG-3′ and reverse: 5′-TGGCATGGACTGTGGTCATG-3′) served as reference gene. Protein levels of ER in basal and transfected MCF-7 cells were investigated by Western blot analysis. Thirty μg of cell lysates were electrophoretically separated and blotted onto a PVDF membrane (Millipore, Billerica, USA) which was subsequently incubated with antibodies specific for ER (Thermo Scientific) and GAPDH (Santa Cruz, Heidelberg, Germany) overnight. Detection of the proteins was carried out using peroxidase-conjugated secondary antibodies (Dako, Glostrup, Denmark) and the chemiluminescence ECL detection solution (Sigma-Aldrich St.Louis, Missouri, USA).

### Apoptosis assay and flow cytometry

Transfected (24 hour transfection) MCF-7 cells (on a 6-well plate) were treated with 6 μM of the topoisomerase I inhibitor camptothecin (Biovision, Milpitas, USA) for 4 hours. Twenty-four hours after induction of the camptothecin-mediated apoptosis, the cells were stained for flow cytometry using the FITC Annexin V Apoptosis Detection Kit (BD Biosciences, San Jose, California, USA) according to the manufacturer`s instructions. The cells were then analyzed on a FACS CantoII flow cytometer (BD Biosciences).

### MTT assay

To measure the cell proliferation, non-transfected and transfected cells were incubated with 20 μL 5 mg/ml MTT (thiazolyl blue tetrazolium bromide, Sigma-Aldrich) in PBS on a 96-well plate at 37°C for 3 hours. Following incubation, the cells were lysed with lysis buffer (4 mM HCl, 0.1% NP40 in isopropanol). A microplate reader (Tecan, Männedorf, Switzerland) was used to measure the OD values at 540 nm. Each experimental group contained three replicate wells, and the experiment was repeated three times.

### Cell migration and invasion assay

Cell migration and invasion were measured using 8-μm pore uncoated or BME (basement membrane extract)-coated transwell inserts, respectively (Trevigen, City of Gaithersburg, Maryland, USA). Briefly, 8 h after transfection with 20 nM mimic, 100 nM inhibitor of *miR-107*, MCF-7 and MDA-MB-231 cells were grown in starvation medium (DMEM with 0.5% FCS and 2 mM L-glutamine) for 24 h prior to their detachment. Cells were resuspended in 400 μL starvation medium, and 100,000 cells per well were added to the top chamber. Medium containing 10% FCS was added to the bottom chamber, and cells were allowed to migrate and invade for 24 h at 37°C. Inserts and wells were washed with 1x washing buffer (Trevigen), and migrated or invaded cells were incubated in 500 μL dissociation buffer containing 0.83 μg Calcein-AM (Trevigen) for 1 h. Fluorescence (excitation: 485 nm, emission: 535 nm) was measured by the LB 940 Mithras microplate reader (Berthold Technologies, Bad Wildbach, Germany). In order to refer relative fluorescence units (RFU) to cell number, separate standard curves for each cell line were performed. On a 24-well plate, a serial dilution of 50,000, 25,000, 12,500, 6,250, 3,125 cells per well was carried out in triplicate. Fluorescence was measured after 1 hour incubation using 500 μL dissociation buffer containing 0.83 μg Calcein-AM (Trevigen). Mean values were plotted for standard curve and the line equation of the trend line was used to calculate the number of migrated or invaded cells.

### Statistical analysis

The statistical analyses were performed using the SPSS software package, version 18.0 (SPSS Inc. Chicago, IL). Because of the skewed distribution of the miRs concentrations, differences in group levels for nonparametric comparisons were bivariately assessed by univariate analyses of the Mann–Whitney-U test of two independent variables and Wilcoxon test of two dependent variables. Diagnostic power of the miRs was analyzed by receiver operating characteristic (ROC) curves. Areas under the curves (AUC) were calculated, assuming nonparametric distribution. Additionally, univariate binary logistical regression was carried out for analyzing combined miRs with the clinicopatholgical data or combined hormone receptor statuses with the variables. For cell lines, the one-way ANOVA with Dunnett and Tukey test was used for all pairwise comparisons that correct for experiment-wise error rate. Missing data were handled by pairwise deletion. A *p*-value <0.05 was considered as statistically significant. All *p-*values are two-sided. Due to the explorative nature of the study no formal adjustment for multiple testing was performed.

## SUPPLEMENTARY MATERIAL, FIGURES AND TABLE


