# Supplementary material for: Aberrant plasma levels of circulating miR-16, miR-107, miR-130a and miR-146a are associated with lymph node metastasis and receptor status of breast cancer patients
Source: Oncotarget. 2015 Apr 19;6(15):13387–401. doi: 10.18632/oncotarget.3874 (PMC4537022; doi:10.18632/oncotarget.3874)
Supplement: Supplementary file 1 [file oncotarget-06-13387-s001.pdf]

Aberrant plasma levels of circulating *miR-16, miR-107, miR-130a* and *miR-146a* are associated with lymph node metastasis and receptor status of breast cancer patients

Supplementary Material

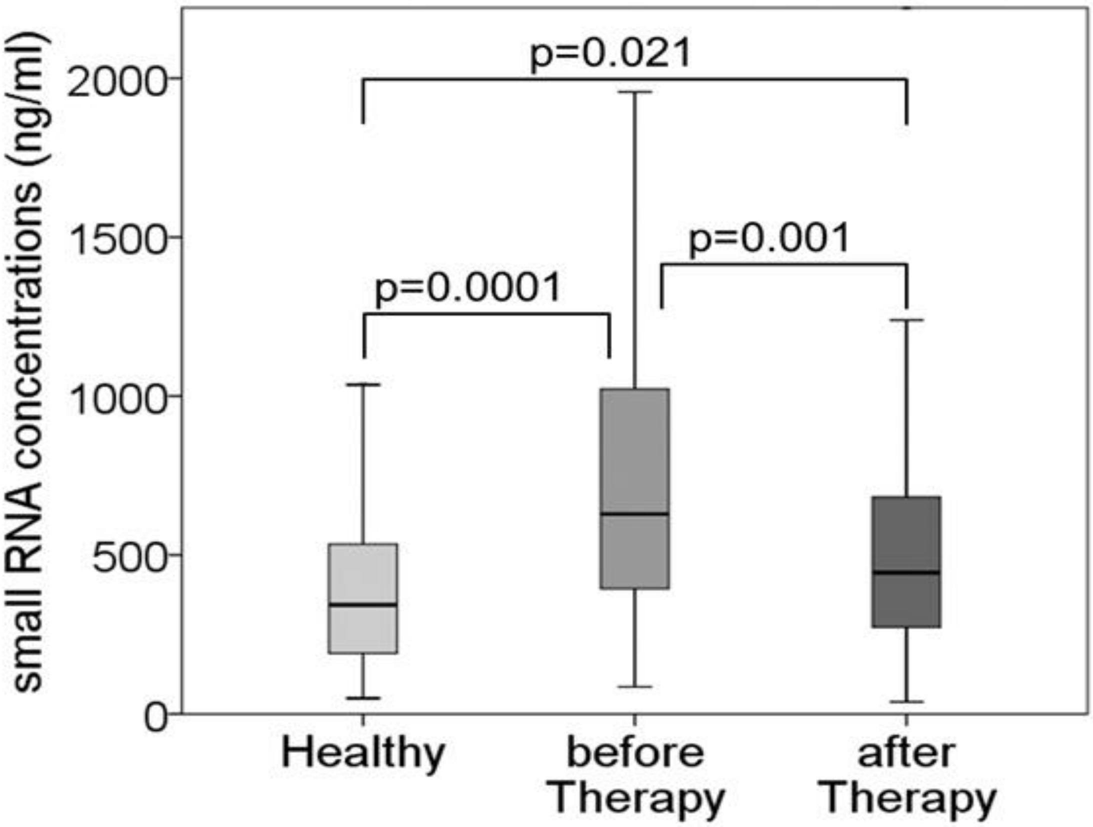

Supplementary Figure S1: Plasma levels of circulating total small RNA in breast cancer patients before and after chemotherapy.

The box plot compares the total small RNA concentrations in the plasma of healthy women (n=46) with breast cancer patients before (n=111) and after chemotherapy (n=111). As determined by the Mann-Whitney-U and Wilcoxon test, the significant p-values of the statistical evaluations are indicated.

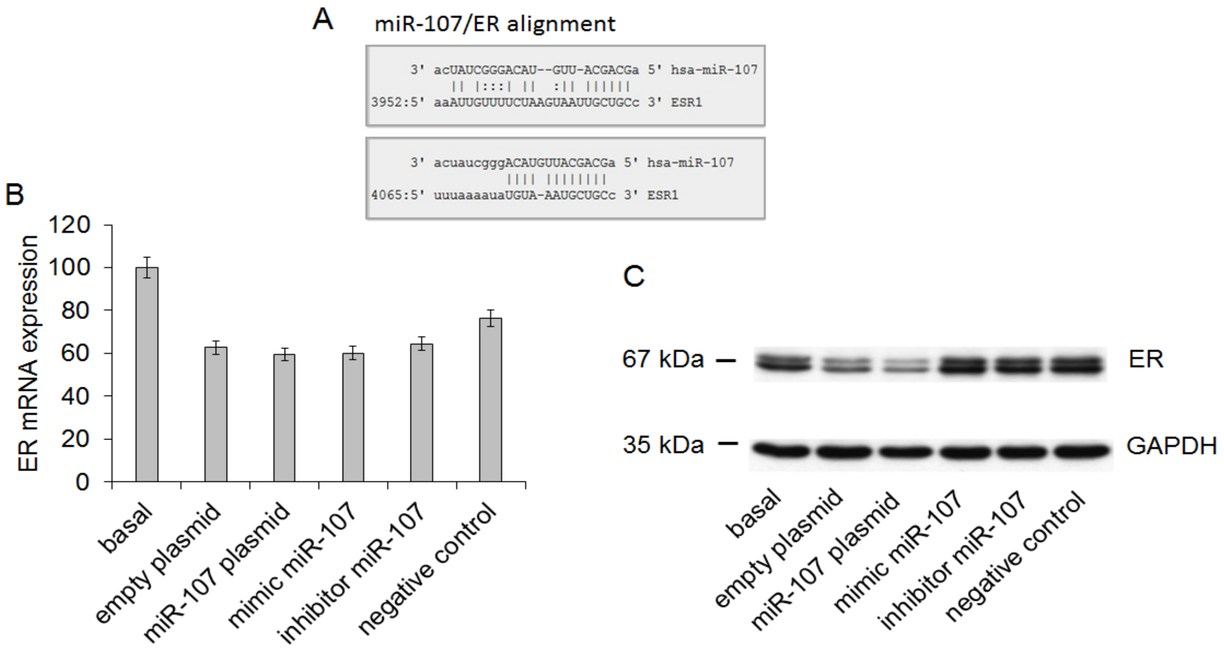

Supplementary Figure S2: *MiR-107* does not affect the protein expression of ER.

The alignment of *miR-107*/ER indicates the two putative binding sites for *miR-107* in the 3`UTR of ER **(A)**. ER mRNA **(B)** and protein **(C)** levels in MCF-7 cells were analysed by real-time PCR and Western blot, respectively. GAPDH signals of the Western blot served as a loading control. ER mRNA and protein levels in MCF-7 cells untreated (basal) or transfected with an empty expression plasmid, a plasmid encoding for *miR-107*, mimic or inhibitor of *miR-107*, or scramble miR (negative control). A representative gel of three independent experiments is shown, all experiments were performed in duplicate.

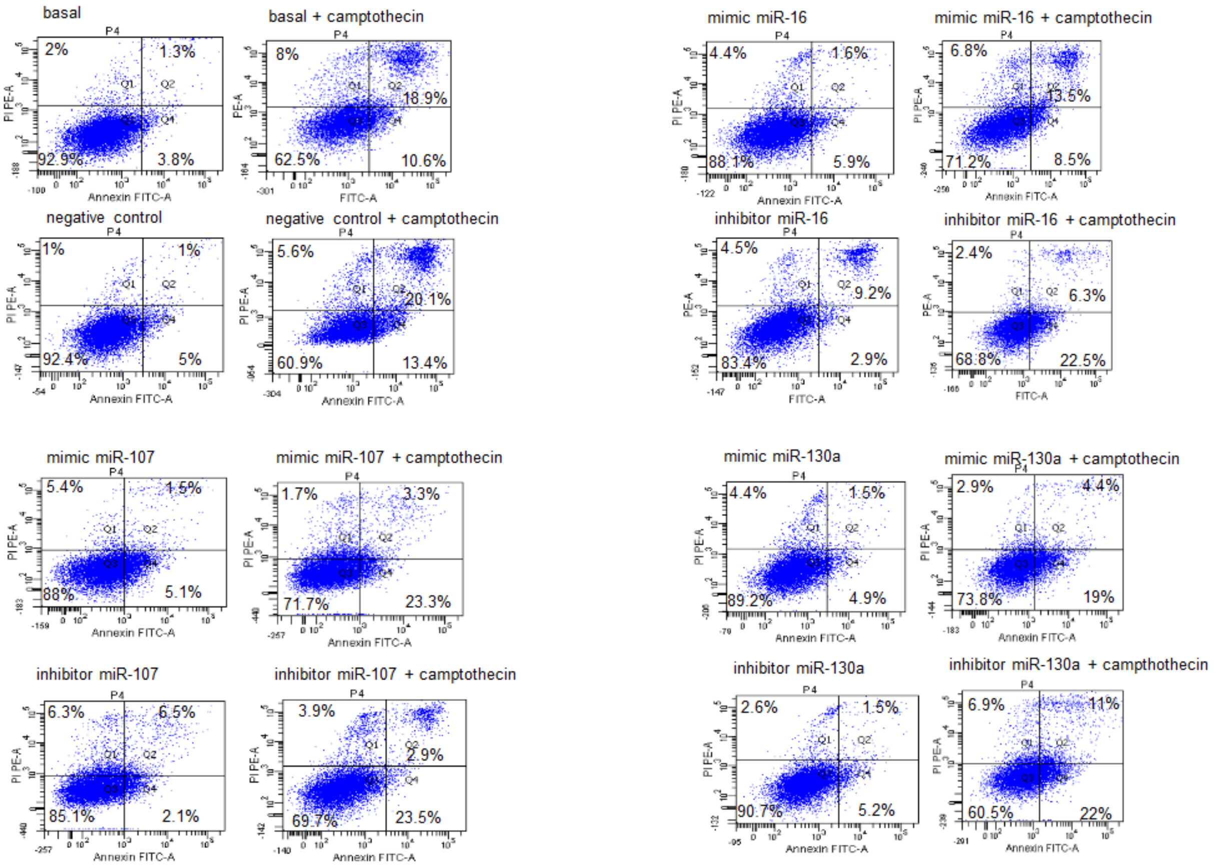

Supplementary Figure S3: *MiR-16, miR-107* and *miR-130a* cannot protect cells against induced apoptosis.

MCF-7 cells were transfected with mimics and inhibitors of *miR-16, miR-107* and *miR-130a* or scramble miR (negative control). Apoptosis in non-transfected and transfected cells was induced by the topoisomerase I inhibitor camptothecin. For FACS analyses on a FACS CantoII device, cells were labeled with annexin-V-FITC and propidium iodide. Cell fragments, positive for only propidium iodide, can be found in the upper left corner (Q1). Late apoptotic as well as necrotic cells can be found in the upper right corner (Q2), since they are positive for annexin and propidium iodide. Living cells, negative for annexin and propidium iodide, can be found in the lower left corner (Q3). Early apoptotic cells, positive for annexin, are located in the lower right corner (Q4). The size for each population (%) is given in the corresponding area.

A) miR-16

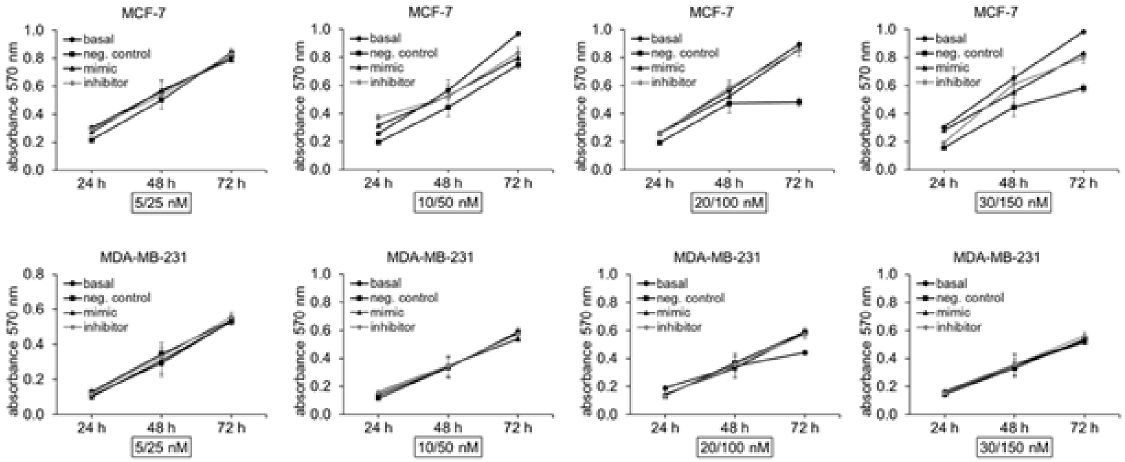

B) miR-107

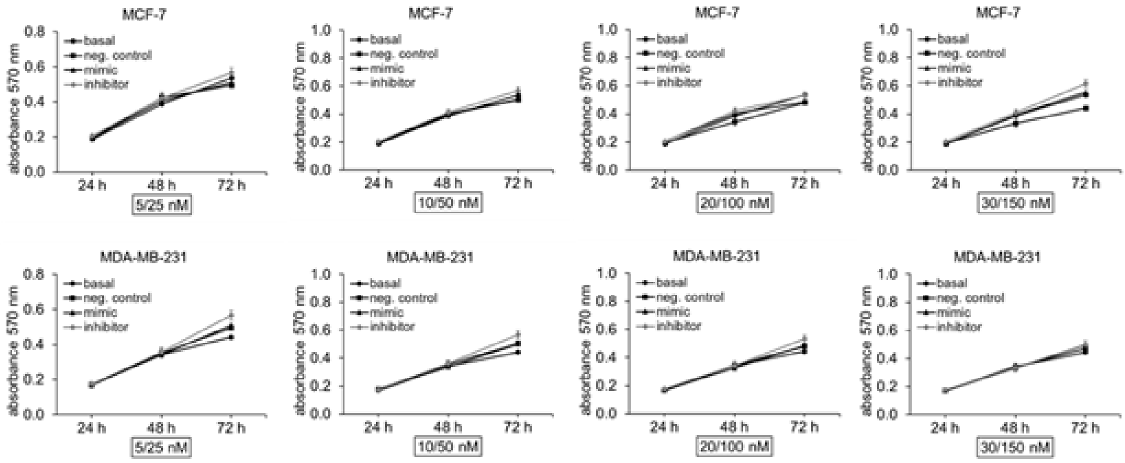

C) miR-130a

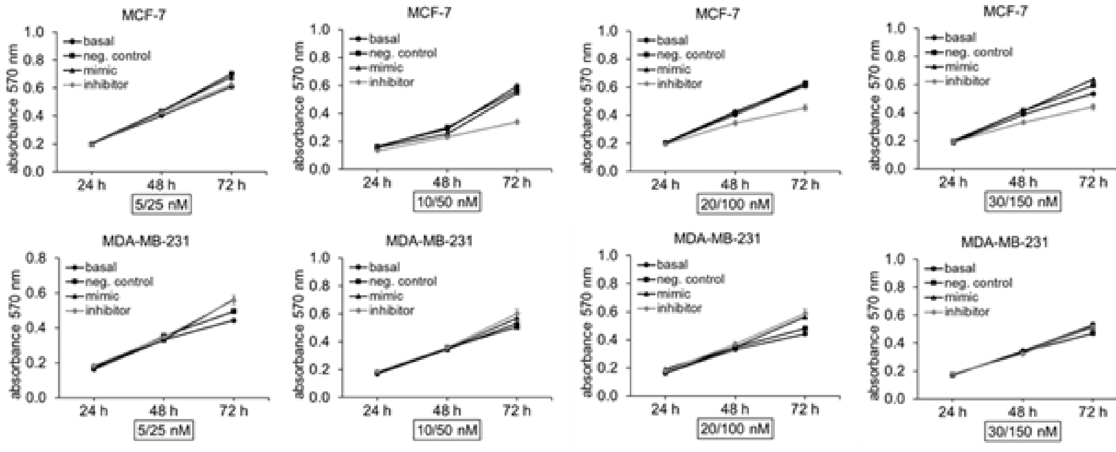

Supplementary Figure S4: Proliferation of MCF-7 and MDA-MB-231 cells transfected with different concentrations of mimics and inhibitors of *miR-16*, *miR-107* and *miR-130a*.

MCF-7 and MDA-MB-231 cells were transfected with mimic or inhibitor of *miR-16* ***(A)****, miR-107* ***(B)*** and *miR-130a* ***(C)*** using four different concentrations 5/25 nM, 10/50 nM, 20/100 nM and 30/150 nM for mimics and inhibitors, respectively. Transfection with scramble miR served as a negative control. Cell proliferation was measured after 24 h, 48 h and 72 h incubation with MTT. Reduced cell proliferation in MCF-7 cells due to the Inhibition of endogenous *miR-130a* was observed for the MCF-7 cells transfected with 50 nM, 100 nM or 150 nM. No significant changes on cell proliferation were observed for *miR-16* and *miR-107* in both cell lines.

Table S1: Associations of miR levels with clinicopathological risk factors of patients before chemotherapy.

Parameters	n (%)	small RNA		miR-16		miR-27a		miR-107		miR-130a		miR-132		miR-146a	
**Breast cancer patients**								Median, Mean, (95% CI) pvalue							
**Total**	111 (100)	647, 834		904, 3179		64, 405		0.4, 1.5		15, 97		2, 9		79, 490	
**Age** (range 33-76 years)	56 years	(703-975)		(1983-4374)		(145-665)		(0.6-2.4)		(19-176)		(3-15)		(73-908)	
**Tumor stage**			0.423		0.338		0.887		0.817		0.575		0.607		0.870
pT1	59 (53)	600, 819		904, 3696		60, 369		0.4, 1.3		10.3, 61.2		2.1, 11		78, 361	
		(616-1021)		(1758-5635)		(95-644)		(0.6-2.1)		(19.6-102)		(0.7-21)		(74-649)	
pT2-4	52 (47)	711, 863		762, 2568		69, 447		0.4, 1.8		15.5, 140		2.2, 6.5		78, 642	
		(679-1046)		(1259-3877)		(-31-924)		(0.1-3.6)		(-27-307)		(1.4-11.5)		(-218-1502)	
**Lymph node metastasis**			**0.043**		**0.019**		0.093		**0.036**		**0.027**		0.089		**0.047**
N0	71 (64)	737, 889		1633, 3657		94, 493		0.4, 1.9		21, 109		3, 12		104, 622	
		(719-1059)		(2108-5206)		(99-887)		(0.6-3.2)		(-5.8-224)		(2.6-21)		(-17-1261)	
N1-3	40 (36)	481, 745		490, 2286		43, 240		0.2, 0.8		8, 75		1.4, 3.8		43, 245	
		(510-980)		(385-4186)		(93-387)		(0.3- 1.4)		(-2.2-153)		(1.9-5.9)		(87-402)	
**Grading**			0.233		0.269		0.275		0.138		0.383		0.084		0.237
II	40 (36)	558, 732		555, 2152		52, 171		0.3, 0.6		15, 24		1.4, 3.7		54, 155	
		(537-926)		(968-3336)		(84-258)		(0.3-1)		(14-34)		(1.5-5.8)		(83-226)	
III	71 (64)	652, 902		1071, 3708		225, 523		0.4, 2		13, 136		2.5, 12		96, 663	
		(716-1089)		(1951-5466)		(121-926)		(0.6-3.4)		(13-259)		(2.5-21)		(13-1314)	
**ER status**			0.595		0.321		0.252		**0.035**		0.542		0.082		0.095
positive	50 (45)	697, 887		717, 2455		59, 430		0.1, 1.5		27, 125		1.8, 6.2		40, 620	
		(661-1113)		(894-4016)		(-58-919)		(-0.2-3.3)		(-42-292)		(1-11)		(-258-1498)	
negative	61 (55)	623, 799,		2569, 3770		69, 984		0.4, 1.5		15, 75		2.5, 11		99, 385	
		(630-969)		(1984-5555)		(115-653)		(0.8-2.3)		(24-126)		(1.1-22)		(100-670)	
**PR status**			0.108		0.816		0.749		0.748		0.306		0.340		0.963
positive	35 (32)	771, 976,		859, 4049		63, 774		0.3, 2.4		10, 164		3.2, 19		90, 1018	
		(696-1256)		(1213-6886)		(-54-1602)		(-0.3-5.1)		(-80-408)		(0.03-39)		(-333-2370)	
negative	76 (68)	566, 777		904, 2784		65, 237		0.4, 1.1		38, 67		1.5, 4.3		76, 251	
		(622-931)		(1569-4000)		(152-323)		(0.6-1.6)		(27-108)		(3-5.7)		(155-347)	
**HER2 status**			0.643		0.910		0.934		0.058		**0.010**		0.193		**0.025**
positive	15 (14)	584, 1018		1370, 2854		71, 934		0.95, 4.6		43, 331		3.6, 13		176, 1703	
		(469-1567)		(88-5620)		(-746-2615)		(-1.5-11)		(-238-899)		(-4-30)		(-1331-4737)	
negative	96 (86)	649, 810		838, 3230		63, 320		0.3, 1		12, 60		1.8, 8.4		67, 297	
		(674-946)		(1899-4562)		(144-497)		(0.6-1.5)		(24-97)		(1.8-15)		(112-482)	
**Triple neg.**			0.176		0.421		0.720		0.331		0.683		0.916		0.430
Triple neg	47 (42)	572, 747		1117, 3032		66.2		0.4, 1.1		15, 64		1.9, 4.3		93, 260	
		(555-939)		(1606-4458)		(124-322)		(0.6-1.5)		(9-119)		(2.7-6)		(134-387)	
other receptor	64 (58)	720, 906		767.33		61, 538		0.3, 1.9		13.1		2, 12		64, 658	
statuses		(714-1098)		(1465-5108)		(91-985)		(0.4-3.4)		(-10-254)		(2-23)		(-63-1380)	
**Healthy women**			**0.0001**		**0.0001**		**0.039**		0.814		0.065		**0.020**		0.177
**Total**	46 (100)	343, 417		300, 483		52, 60		0.3, 0.6		9, 14		1.4, 1.7		60, 85	
**Age** (range 50-85 years)	63 years	(326-508)		(294-672)		(41-79)		(0.2-1)		(9-18)		(1.1-2.3)		(58-112)	

Significant p values in bold; p values as determined by Mann-Whitney-U test. *Confidence Interval, CI. Median, Mean of relative values.
